# Organelle membrane derived patches: reshaping classical methods for new targets

**DOI:** 10.1038/s41598-017-13968-y

**Published:** 2017-10-26

**Authors:** George Shapovalov, Abigaël Ritaine, Gabriel Bidaux, Christian Slomianny, Anne-Sophie Borowiec, Dmitri Gordienko, Geert Bultynck, Roman Skryma, Natalia Prevarskaya

**Affiliations:** 1 Inserm U1003, Equipe Labellisée par la Ligue Nationale Contre le Cancer, Université de Sciences et Technologies de Lille (USTL), F-59655 Villeneuve d’Ascq, France; 2 Laboratory of Excellence, Ion Channels Science and Therapeutics; Université Lille I Sciences et Technologies, Villeneuve d’Ascq, France; 3 Laboratoire INSERM U1060, CarMeN Laboratory, Claude Bernard Lyon 1 University, 8, avenue Rockfeller, F-69373 Lyon, France; 4 KU Leuven, Laboratory of Molecular and Cellular Signaling, Department of Cellular and Molecular Medicine, Herestraat 49, BE-3000 Leuven, Belgium

## Abstract

Intracellular ion channels are involved in multiple signaling processes, including such crucial ones as regulation of cellular motility and fate. With 95% of the cellular membrane belonging to intracellular organelles, it is hard to overestimate the importance of intracellular ion channels. Multiple studies have been performed on these channels over the years, however, a unified approach allowing not only to characterize their activity but also to study their regulation by partner proteins, analogous to the patch clamp “golden standard”, is lacking. Here, we present a universal approach that combines the extraction of intracellular membrane fractions with the preparation of patchable substrates that allows to characterize these channels in endogenous protein environment and to study their regulation by partner proteins. We validate this method by characterizing activity of multiple intracellular ion channels localized to different organelles and by providing detailed electrophysiological characterization of the regulation of IP_3_R activity by endogenous Bcl-2. Thus, after synthesis and reshaping of the well-established approaches, organelle membrane derived patch clamp provides the means to assess ion channels from arbitrary cellular membranes at the single channel level.

## Introduction

Intracellularly localized ion channels are known to play important role in various cellular signaling pathways which are often directly involved in the regulation of cellular fate. For example, the Ins(1,4,5)P3 and ryanodine receptor channels (IP_3_R and RyR), with their well-established localization in the ER, regulate various important cellular functions ranging from muscle contraction to the processes of cellular development, motility and death^1–3^. In recent years a growing number of the channel types embedded in the membranes of nucleus, endoplasmic reticulum (ER), mitochondria, mitochondria-associated membranes (MAM), endosomes and lysosomes^4–8^ have been identified, including but not limited to the mitochondrial Ca^2+^ uniporter MCU, K_V_1.3 and 10.1, K_ATP_, TRPM8, TRPML1, TWIK and TASK channels.

The high rate of discovery of intracellular ion channels and the fact that approximately 95% of the membranes are intracellular, have exposed these channels as an important class of proteins, more numerous than those expressed in the plasma membrane (PM). However, despite their abundance, intracellular ion channels remain poorly characterized at a single channel level. Contrary to the ion channels embedded in the plasma membrane, the intracellular localization of these proteins significantly complicates the application of classical electrophysiological techniques, such as patch clamp. The publications that report on electrophysiological properties of these channels often utilize planar lipid bilayer experiments. Yet, this well established technique has certain limitations: large electrical capacitance of the bilayer resulting in low acquisition bandwidth and the nessesity of purification of the investigated channel protein, compromising the study of regulation of ion channels by their native protein partners^9,10^. In order to overcome the latter, some studies, as early as 1980s, employed fusion of small vesicles of a limited number of intracellular membranes with planar lipid bilayer (e.g.^11^). Although this has not become a universal trouble-free approach, it prompted more recent attempts at perfecting the protein-insertion protocols, such as^12^.

Ironically, a series of much earlier publications, dating back 20–30 years, some even prior to the advent of the classical patch clamp, hinted at approaches that potentially could resolve the above issues: collecting different membrane fractions followed by fusion of this material with sufficiently large liposomes prepared via some pre-established routines, such as enlarging small unilamellar vesicles (SUVs) by subjecting them to freeze-thaw or dehydration-rehydration cycles^13,14^, or by inducing blister formation in lipids deposited directly on the chamber floor^15^. Separately we should note the use of *in vitro* transcription-translation system by Sukharev *et al*.^16^, who first showed the possibility to incorporate fractionated membranes, containing bacterial mechano-sensitive channels of large conductance (MscL), into liposomes and then successfully patch them. These authors went on to expand this work to *in vitro* production of MscL. The publication by Jonas *et al*.^17^, employing a state of the art approach also merits a separate notice – the “pipette in a pipette” technique, that utilizes a double-seal formation with first, wider pipette clearing access for the 2^nd^ thinner pipette into intracellular compartments. The relatively tedious manipulations involved and the need for two independent micro-manipulators instead of one prevented, however, a wide adoption of this approach. Summing up, these prior publications presented interesting protocols and encouraged the study of the intracellular channel activity, although often requiring tedious manipulations, mirrored by author’s own experience^18,19^. These early studies set a stage for further development necessitating additional work to systematize them and formulate more universal approaches.

An alternative approach addressing the study of intracellular ion channels involved patching intact organelles, for which an isolation procedure had been established. However, after almost 30 years this research is still limited to the nuclei of selected cell lines, mitochondria, enlarged lysosomes and a few plant organelles, such as vacuolar, thylakoid and chloroplast membranes^20–28^. Recent technical advances have allowed to streamline and standardize the manipulations relating to patching these organelles, as has recently been described, for example, by Schieder *et al*.^29^. However these advances have not yet encouraged patching of other organelle types.

Moreover, attempts to compare the activity of ion channels recorded in intact organelles and those purified and reconstituted in lipid bilayer, frequently show significant discrepancies. For example, a native IP_3_R channel in nuclear envelope typically exhibits drastically different gating properties, with significantly higher P_open_ than that of the purified protein reconstituted in a planar lipid bilayer^10^. As it is pointed above, these differences can be attributed to the loss of protein-protein interactions in the lipid bilayer preparations, not to mention variations in the lipid composition as well as possible post-translational modifications. Thus, rigorous study of the arbitrarily localized intracellular ion channels and their regulation by partner proteins (such as in the case of the IP_3_R s and Bcl-2 in ER and MAMs; see above) is still beyond the scope of the approaches readily available to broad scientific community.

Here, we present a novel approach, organelle membrane derived (OMD) patch clamp, which allows electrical recording of the activity of a single ion channel localized in an arbitrary sub-cellular fraction, such as ER-, mitochondria- or liposome-enriched membranes, without compromising its interaction with endogenous partner proteins. The ability to preserve the endogenous protein environment in isolated intracellular membranes has the distinct advantage for study of protein-protein interactions in signaling cascades recruiting ion channels. Furthermore, this method does not require elaborate organelle extraction procedures and lab-specific know-how and, hence, is not limited to particular organelles. At the same time, it provides an opportunity to modify the channel micro-environment, if necessary, to assess diverse protein interaction models. To highlight the universality of our approach, we demonstrated the recordings of the activity of multiple ion channels derived from different organelles of various cell types, namely, the endogenous IP_3_R and RyR channels from the ER of HEK293 (human endocrine kidney), LNCaP (human prostate cancer) and WEHI7.2 (murine thymic lymphoma, an immature T-cell line) cells^30^, and TPC channels from lysosomal fraction of HEK293 cells. Finally, we showed the on-line modulation of the activity of the intracellular ion channel (IP_3_R) localized in the large amorphous and, hence, non-extractable organelle (ER) by the endogenous membrane-bound partner protein (Bcl-2).

## Results

### Description of the approach

**Figure 1:**
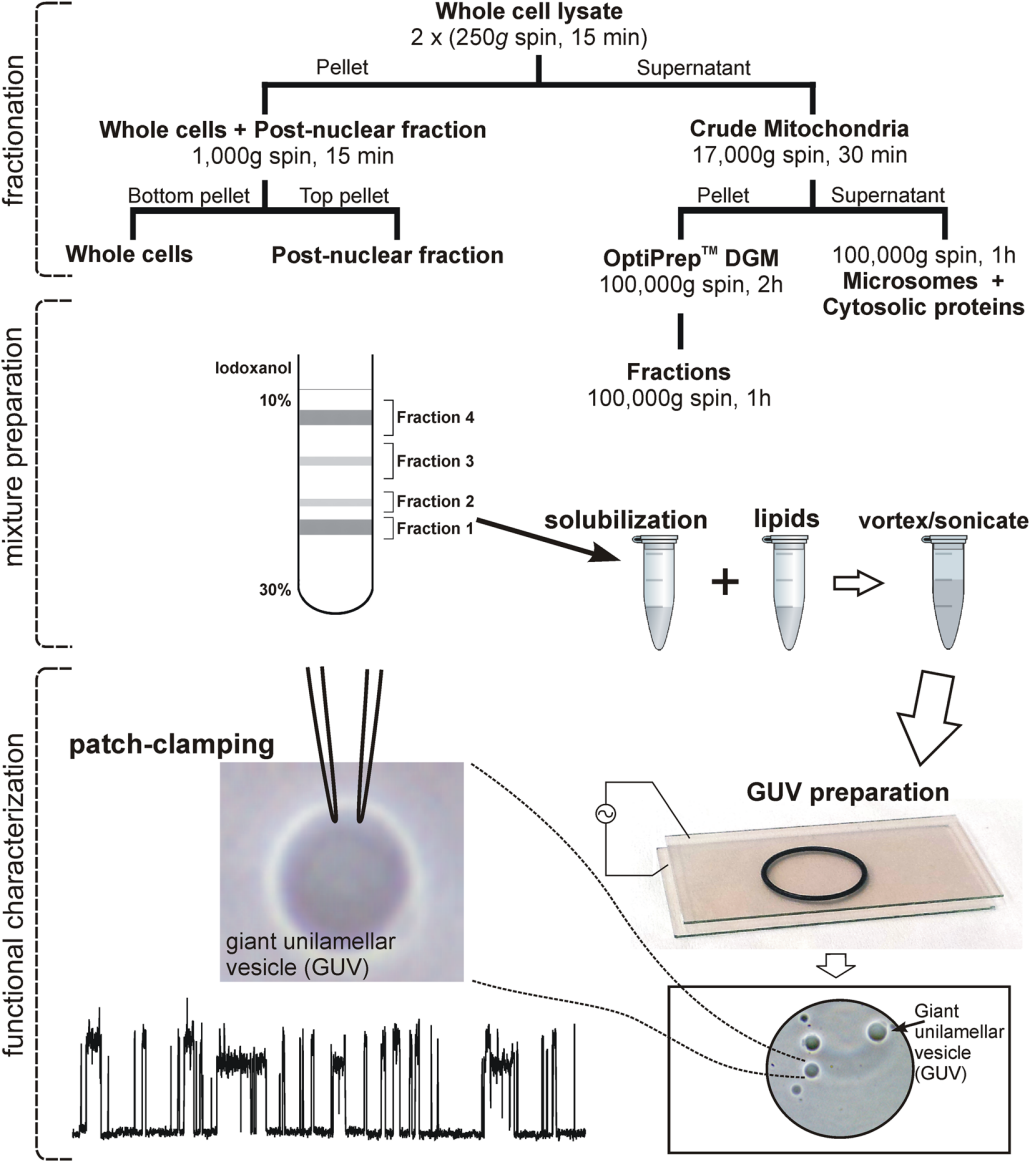
Principal diagram of the approach. The diagram illustrates fraction isolation and preparation of patchable substrates. Top (fractionation): a flowchart presenting example avenues leading to preparation of various types of membrane fraction extracts. Middle (mixture preparation): an illustration of the process of the preparation of the fraction extract/lipid mixture suitable for - bottom (functional characterization) – containing an illustration of the giant uni-lamellar vesicle (GUV) preparation and patch-clamping.

At its core, the proposed approach combines established biochemical isolation methods of various organelles with the production of patchable vesicles containing endogenous proteins extracted from specific subcellular membrane fractions. The principal steps comprising this approach are outlined in Fig. 1. The technique employs as a first step the isolation of either a specific membrane fraction or a collection of cell membranes that are intended for further functional characterization. The collected fraction(s) are optionally combined with additional lipids, allowing one to achieve the suitable per-patch ion channel density and/or to provide certain degree of control over lipid environment. The introduction of additional proteins of interest, or performing steps towards purification or concentration of particular protein can also be carried out if necessary at this stage, thereby expanding the versatility of this approach. Next, the resulting mixtures are employed in the preparation of patchable substrates - giant unil-amellar vesicles (GUVs). The activity of channel proteins reconstituted in such vesicles can then be studied using either a conventional patch-clamp setup, or using an automatic patch-clamp system, such as PatchXpress systems by Molecular Devices or Port-a-Patch (that was used to acquire some of the data presented here), or Patchliner by Nanion to name just a few. The biochemical manipulations necessary to isolate membrane fraction are described in more detail below, in the subsections devoted to validation of this technique on preparations from specific organelles, such as ER and lysosomes as presented here. However any established and validated isolation protocol can be applied at this stage. It is important to note, that the isolation of intact organelles is not necessary, as only the pellet containing the lipid/protein mix corresponding to target organelle(s) is needed for further manipulations, which we describe in detail further on. This significantly expands the range of applicable extraction procedures, allowing the isolation of a much greater selection of intracellular organelles, as well as the use of simpler extraction protocols.

Following the isolation of specific membrane fraction, the collected pellet is split in two, with one part re-suspended in RIPA buffer for further biochemical control via Western blot, whilst another is set aside for the vesicle preparation. This part of the pellet selected for vesicle preparation is transferred to a glass vial and is vacuum-dried for a short period (typically 5–15 min), in order to remove any aqueous component. This is followed by the gradual addition of 5:1 CHCl_3_:MeOH mixture to yield a saturated solution of isolated fraction mixture. Typically, 1 ml of solvent is necessary to reliably solubilize the fraction mixture produced from a few million cells, depending on organelle ubiquity and isolation protocol efficiency. The isolated and solubilized fractions are then optionally combined with the suitable lipid combination, such as 5 mM DPhPC and 0.5 mM cholesterol, in order to dilute the fraction mix, should too many channels per patch be observed during data acquisition. Typically, an initial combination is attempted at the 1:1 ratio of fraction and 5 mM lipid mixture in the case of cells over-expressing proteins of interest. This ratio can then be adjusted as necessary, until the desired per-patch channel density is observed during electrophysiological characterization.

The collection and solubilization of membrane fractions is followed by the preparation of giant uni-lamellar vesicles, using the electro-formation technique (see e.g.^31^ for the protocol description using custom-made system and^32^ for the use of commercial setup). Either a custom-made vesicle formation chamber or one of the commercially available setups that have recently appeared on market, such as Vesicle Prep Pro by Nanion Technologies (Germany), can be used. In short, the prepared fraction/protein mixture is dried on the ITO glass slide, enclosed in an o-ring and then rehydrated by the addition of a sucrose or sorbitol-based medium, with the total osmolarity matching that of the solutions used during patch clamping (typically 300 mM for further recordings in physiologically-matching conditions). This preparation is then covered by another ITO glass slide and loaded into either a commercial apparatus or a custom-made stimulation chamber and the GUVs are produced by applying an AC stimulation of 3 V at 5 Hz frequency and T = 30 C for 2 hours. The GUVs produced are collected immediately after formation and can be used for patch clamping right away, or stored for up to a week at 4 °C.

Classical ion channel solubilization protocols call for storing the proteins in detergent solutions (see e.g.^18,33,34^). However, detergent-based solubilization requires additional steps to remove detergent following the lipid addition, thus complicating manipulations. In practice, no difference between different solubilization approaches (either using the approach presented here, or following the protocol used by one of the authors in the past, see^18^) was observed during the acquisition of the functional data presented below. It should be noted though, that if detergent solubilization of the proteins is used, care should be taken to select appropriate detergents. Thus, RIPA buffer, commonly used to store proteins for further characterization via Western blot, is known to denature proteins. Instead a milder detergent, such as CHAPS is normally preferred to store purified or isolated channel proteins^18,34^.

### Use case 1: activity in isolated ER membrane fractions

To illustrate the application of this approach, we applied the steps outlined above to isolate, prepare vesicles and patch the ER containing fractions from the HEK293 and LNCaP (human prostate cancer) cells. The particular isolation protocol of the ER-containing membrane fraction was based on the well-established procedures for organelle enrichment^35^, outlined in Fig. 1. The top portion of Fig. 1 presents a flowchart of possible manipulations that lead to the collection of post-nuclear fractions, mitochondria, dense ER membranes, Golgi vesicles or light smooth ER membranes (microsomes). In short, HEK293 or LNCaP cells were collected in a phosphate buffer saline solution (PBS), pelleted in 50 ml tubes and homogenized in ice-cold buffer as described. The non-disrupted cells and post-nuclear fraction were sedimented and separated by the series of mild centrifugations. A further separation of mitochondria and associated membranes could be carried out by stronger centrifugation, separating the MAM fraction and the rest of the subcellular membranes, as described. Subsequent fractionation of vesicles and membranes was performed with an Iodixanol (OptiPrep^TM^) gradient^36,37^ with minimal physical disruption to limit degradation of organelle membranes, to allow us to collect specific organelles and vesicles for further processing^38^.

**Figure 2:**
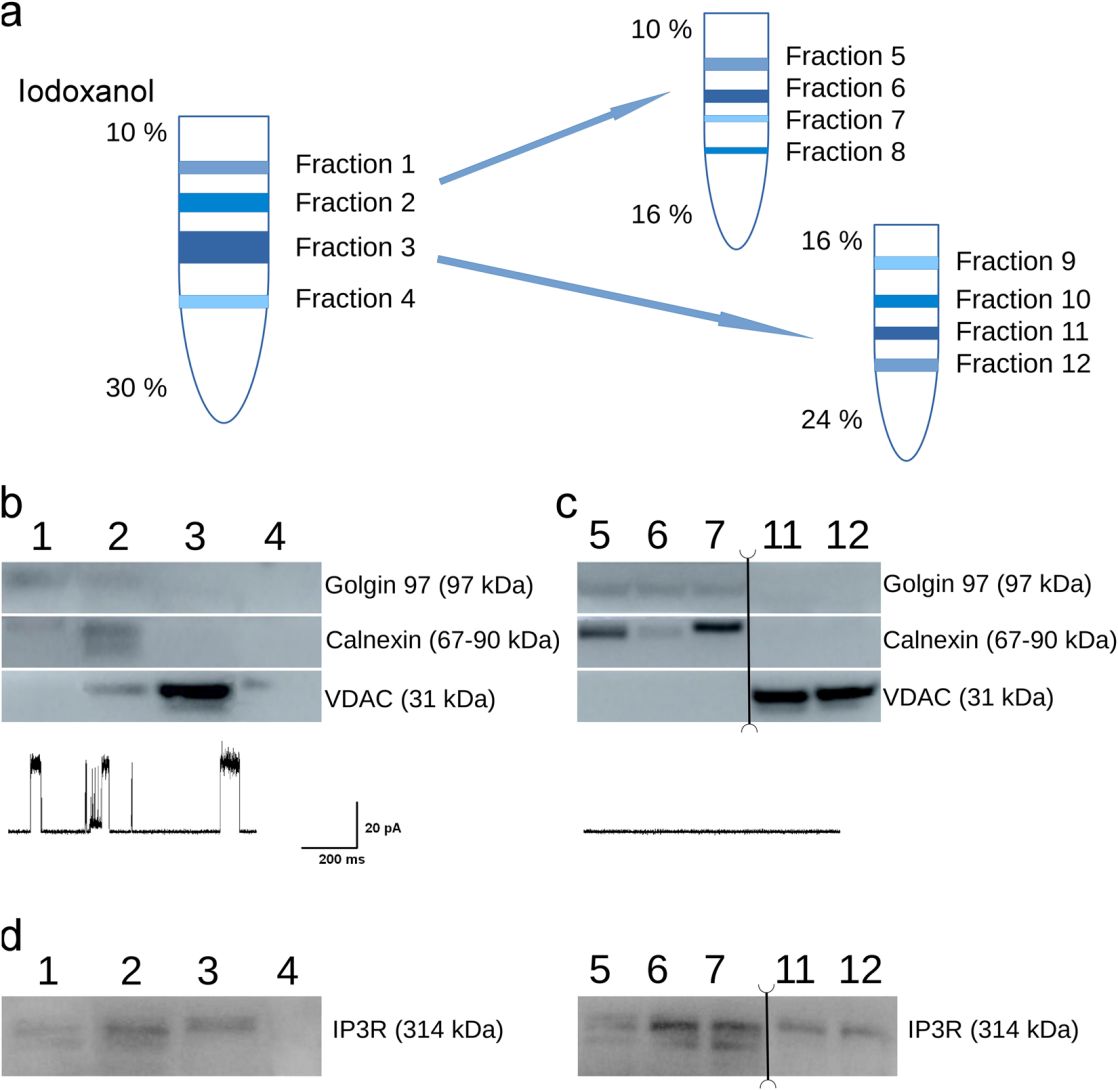
Bichemical characterization of the isolated fractions and localization of the IP_3_R protein. (**a**) A principal diagram of gradient centrifugations yielding crude and better isolated fractions following single and double-gradient centrifugation, showing fraction numbers. (**b,c**) Western blot characterization of organelle-specific markers in the isolated fractions following single- (**b**) and double- (**c**) gradient isolation of subcellular membrane fractions. ER membranes are reported by the detection of calnexin (upper panels), while Golgi apparatus and mitochondria are characterized by the detection of golgin-97 and voltage-dependent anion channel 1 (VDAC1) (middle and lower panels), respectively. Note that, following the double-gradient isolation, Fractions 8, 9 and 10 did not contain sufficient amounts of protein and were thus not characterized. Traces below Western blot images indicate presence (**b**) or absence (**c**) of VDAC-like activity in the patches made of GUVs produced from isolated fractions. (**d**) Western blot characterization of the IP3R presence in the isolated fractions following single- (left) and double- (right) gradient isolation protocol. Note the enrichment of IP3R presence in purer ER and MAM sub-fractions following double-gradient isolation. Splices of the double-gradient gel, where two non-adjacent parts of the same gel with the same exposure time have been emerged together, are indicated with vertical lines, between fractions 7 and 11. The original uncut gel can be seen in Fig. S1. Experiments were reproduced 4 times independently.

To demonstrate the subcellular origin of the isolated fractions and to attribute them to specific intracellular membranes, a small part of the material produced during the fractionation was separated and used to test for the presence of organelle-specific protein markers. The results of such characterization for this validation study, presenting a typical distribution in one out of 5 different procedures of protein fractionation, are shown in Fig. 2. Figure 2a shows a scheme of multiple gradient centrifugation steps, necessary to address the cross-contamination often observed following the most common version of the separation protocol (e.g.^36^), as discussed in more detail below. Figure 2b displays the results of Western blot analyses of the isolated fractions. The ER membranes were reported by the detection of calnexin, while mitochondria and Golgi apparatus were characterized by the detection of voltage-dependent anion channel 1 (VDAC1) and of golgin-97, respectively. Further, the presence of the intracellularly localized ion channel IP_3_R in isolated subcellular fractions was verified by Western blot experiments, using the Pan anti IP_3_R Rbt476 antibody recognizing all three IP_3_R types (a kind gift from Prof. J. Parys^39^). Figure 2d shows the typical distribution of the IP_3_R protein in the isolated fractions, confirming its presence in the ER-enriched fractions.

**Figure 3:**
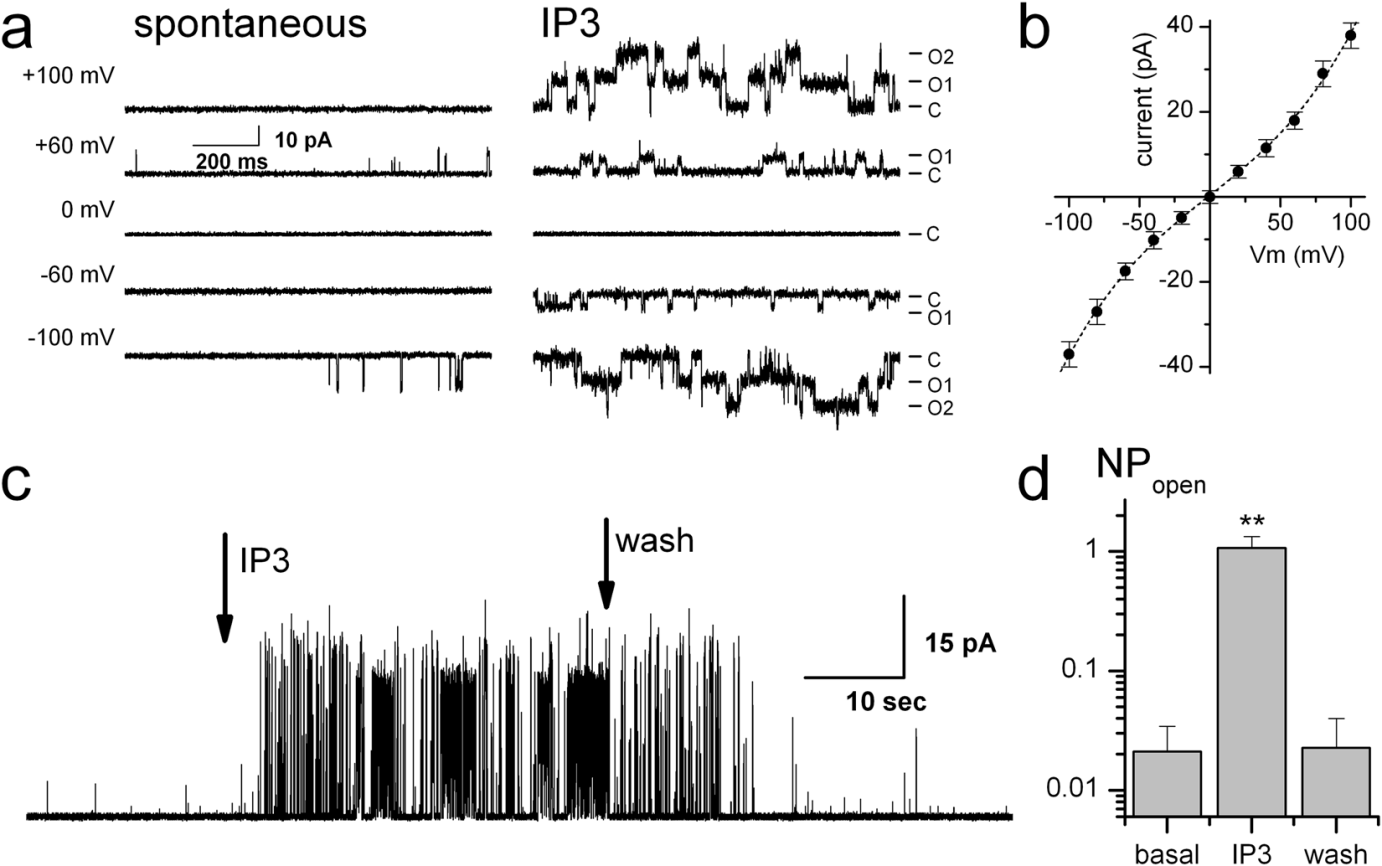
Characterization of IP_3_R activity in whole-membrane extracts from HEK293 and in ER fractions from LNCaP cells. (**a**) Typical basal (left) and stimulated by 10 μM IP3 (right) activity at the series of applied potentials indicated on the left. C, O1 and O2 on the right indicate closed, open states and double openings respectively. (**b**) Typical IV relationship. Note the bend indicating smaller single-channel conductance near zero, characteristic of IP_3_R activity in the presence of Mg^2+^. (**c**) Sample IP_3_R activity showing basal region, followed by increased activity in response to application of 10 μM IP3 and, finally, reduction of activity upon washout. (**d**) Average NPopen in basal, IP3 stimulated and wash regions is summarized in a barplot (mean ± s.e.m.; **denotes significant difference with p < 0.01; n = 7). See also Fig. S2.

Next, we used the isolated material to create and patch GUVs following the procedure outlined above. Fraction-2 (Fig. 2b) was selected for vesicle creation, as better representative of the ER-containing isolate. GUVs were prepared from the isolated fraction material combined with the 10:1 DPHPC/cholesterol lipid combination (5 mM). Initially the mixture with the 1:1 ratio of fraction material to additional lipids was tested. However, such preparations would often yield traces with multiple levels of IP_3_R activity universally present, necessitating further dilution of the isolated fractions. Typically, the mixtures with 1:2 – 1:5 ratios were used, yielding activity of IP_3_R channels in approximately half of the patches. The IP_3_R activity could be readily and reversibly stimulated by the application of 10 μM IP3 at the [Ca^2+^]_i_ of 1 μM, in close correspondence to the known properties of the IP_3_R channel^10^. The acquisition of the IP_3_R activity at various potentials applied across the membrane (Fig. 3a), revealed an IP3-sensitive activity with a mean conductance of 330 ± 11 pS in a symmetric 145 mM KCl solution and demonstrated a characteristic sensitivity^10,22,40^ of the single channel conductance to the presence of Mg^2+^, as shown in Fig. 3b. This characteristic IP3R activity was observed in 37 out of 71 patches made of GUVs prepared from the 1:5 mixtures and included in the original analysis reported here (Fig. 3c,d). However, later, we observed similar activity on a regular basis while carrying out other projects. Similar recordings carried out on GUVs prepared from fraction-1, which exhibited a much lower IP_3_R density, yielded such IP_3_R activity at least 10 times less frequently (only 3 out of 26 patches made of GUVs using 1:1 mixtures; data not shown).

Additionally, to demonstrate the universal character of this technique, which is not limited to observing activity of only a specific ion channel, we also attempted to record RyR activity in the same membrane fractions. The recording conditions were changed to emphasize RyR activity over that of IP_3_R, making use of the differential sensitivity of these channels to Mg^2+^, as described. Typical gating of RyR is shown in Fig. S2, demonstrating such characteristic features^41,42^ as further stimulation of spontaneous activity by 3 mM caffeine, blocking of activity by 1 μM dantrolene and an increase in the channel P_open_ by 1 nM dantrolene. Another illustration of the successful use of this technique can be seen in^6^, where we have characterized the activity of short TRPM8 channel isoforms, which are localized in the ER in prostate cancer cell lines.

### Improving the purity of isolated fractions

Note, that while the procedure described above has allowed a separation of the intracellular material into the fractions that could be attributed to a particular organelle, a noticeable cross-contamination could still be observed, as evidenced by the presence of multiple trace markers in most fractions. Thus Fraction 2 in Fig. 2, that was the best representative of the ER membrane, and was thus used for further characterization, still contained traces of the mitochondria membranes, evidenced by the presence of the VDAC protein (Fig. 2b). Indeed, while patching the GUV preparations made from this fraction, we could observe not only IP3R and RyR activity described above. In approximately 11% of the traces, we could observe VDAC-like activity^43^, as can be seen in the lower panel of Fig. 2b. While it was reasonably easy to differentiate activity of different ion channel species in this preparation, such contamination can significantly complicate the analysis of ion channels whose identity has yet to be firmly established.

To alleviate this complication additional steps can be taken to better separate materials belonging to different intracellular organelles. In this case, to achieve better separation, we extended the protocol by performing multiple gradient centrifugation steps of increasing density resolution. Thus, as illustrated in Fig. 2a, following the 1^st^ gradient centrifugation, the material belonging to Fraction 2 was collected and applied to a 2^nd^ Iodixanol gradient spanning the subrange of the original densities, chosen to include the mean density corresponding to the observed band. In this case, the 2^nd^ gradient of 10–16% was used, following the 1^st^ 10–30% gradient. Figure 2c compares gradient bands and Western blot characterization results obtained after single and double-gradient separation. Note that, as the 2^nd^ gradient contained significantly less per-fraction material, only the bands containing sufficient quantities of total protein are shown. As can be seen, double-gradient separation significantly improved fraction isolation purity by suppressing the cross-contamination observable after a single-gradient separation. Furthermore, only IP3R and RyR, but no VDAC-like activity was observed in 26 patches made to the GUVs prepared from the band collected after the 2^nd^ gradient centrifugation, as illustrated in the lower panel of Fig. 2c.

As can be seen from these results, expanding the standard protocol to include additional steps allows the obtention of a better fraction separation. Thus one can observe a typical balance between effort spent, quantity of collected material and cleanliness of preparation. This presents a classic choice: while it is critical to obtain maximal separation of the intracellular membrane fractions when working with not yet well-characterized ion channels, it may be preferable to collect more material, whilst using a less tedious set of manipulations when working on ion channels whose identity is already well-established.

### Use case 2: isolating and patching lysosomal fraction

To better illustrate the applicability of this approach to studying ion channels in arbitrary organelles, we isolated another organelle fraction, which would however require a different isolation protocol. Studies of lysosomal composition and associated ion channels have been performed for some time now and lysosomal isolation protocols are well established. Moreover, to illustrate the ease of combining various biochemical procedures with vesicle preparation, we opted to use a commercially available lysosome isolation kit, used in publications studying lysosomal ion channels^44^.

**Figure 4:**
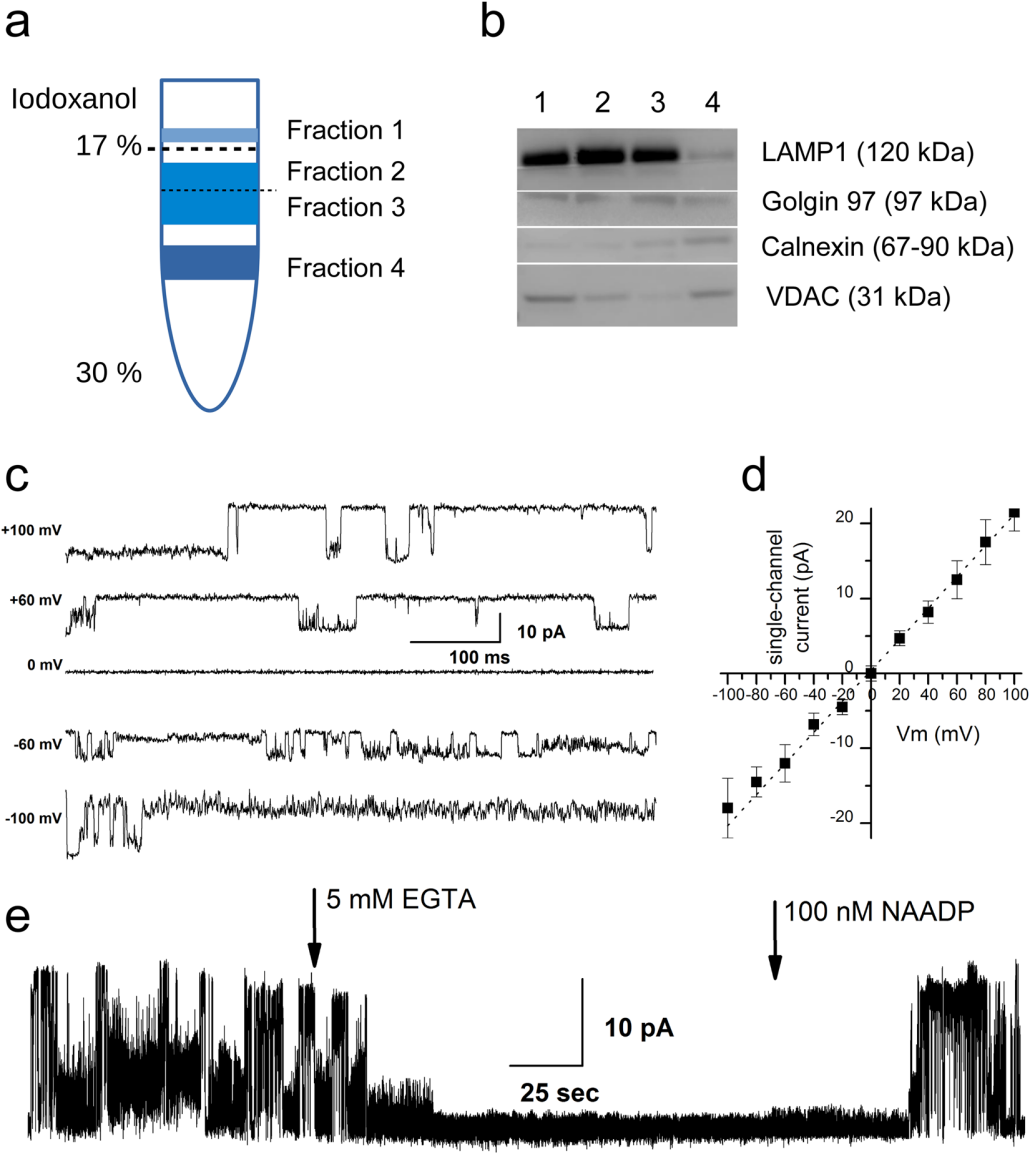
Isolation and single-channel activity in the lysosomal fraction. (**a**) Diagram illustrating the distribution of HEK293 lysate material following the procedure of the lysosome isolation kit protocol and the numbering of the isolated fractions. Lysosomal fraction is positioned in the top-most region of the gradient, directly below the narrow white band of light material that did not enter the Iodixanol gradient. The top edge of the gradient (17%) is indicated by a dotted line and another dotted line below indicates the split of wide lysosomal band into Fractions 2 and 3. (**b**) Distribution of molecular markers in the isolated fractions. (**c–e**) Patch-clamp characterization of TPC2 activity in lysosomal fraction, showing sample IV activity (**c**), IV curve under symmetric 150 mM KCl conditions, yielding representative full-open state conductance of 207 ± 16 pS (**d**) and [Ca^2+^] dependence of TPC2 activity (**e**).

Lysosomes were isolated following the kit manufacturer’s protocol. In short, HEK293 cells were tripsinated, collected, centrifuged to remove media and treated for 2 min with 800 µl of kit Solution A, transferred to a Dounce tissue grinder and lysed. The lysate was transferred to an eppendorf tube and 800 µl of kit Solution B were added and mixed gently. The mix was centrifuged at 500 g for 10 min and the supernatant layered on top of a discontinuous gradient prepared as described, which was then centrifuged at 145,000 g for 2 hours. After the centrifugation, multiple bands could be observed in the gradient, with the lysosomal fraction positioned in the top-most density band corresponding to the 17% Iodixanol density. As previously, multiple fractions were collected as indicated in Fig. 4a, in individual eppendorf tubes and centrifuged at 18,000 g for 30 min. The resultant pellets were split, with small pieces of material set aside for biochemical characterization and the rest was vacuum-dried and solubilized in a chloroform/methanol mixture. As can be seen in Fig. 4b, Fractions 2 and 3, which represent top and bottom halves of the lysosomal fraction band, both contained large amounts of the lysosomal marker protein LAMP-1. Fraction 2 still contained trace amounts of VDAC, probably due to the presence of residual fragments of the top-most fraction of light material, while Fraction 3 was reasonably free of such impurities, although still exhibiting a slight Golgin presence. While the isolated fractions (especially Fraction 3) were significantly richer in lysosomal proteins and contained only trace amounts of ER and MAM-associated marker proteins, a sub-fractionation of the lysosomal band, following multi-gradient isolation, similar to that described above, may be advisable if the presence of such impurities is a major concern in the study attempted.

Next, activity in lysosomal fraction was characterized by preparing and patching GUVs, as above, from the isolated Fraction 3 material directly, without addition of extra lipids. Analysis of the single-channel activity in collected traces has shown, among other things, a presence of the signature currents of the two-pore segment channel 2 (TPC2) proteins, that are known to be endogenously expressed in HEK293 and localized in lysosomes^45^. TPC2 activity, observed in 4 out of 26 patches, could be identified by multiple factors: first, by its characteristically large conductance with observable multiple conductive substates, populated in a voltage-dependent manner (207 ± 16 pS slope conductance, Fig. 4c and d) and further, its activity could be inhibited by removal of free calcium following the application of 5 mM EGTA and later re-established by the application of 100 nM NAADP (Fig. 4e), in close accordance with recent TPC2 characterizations carried out in nuclear patch and lipid bilayer recordings^46,47^.

### Interaction of Bcl-2 and IP3R is preserved in the patchable substrates

Finally, we show that the proposed OMD patch-clamp approach preserves endogenous channel complexes in association with their endogenous accessory proteins present in specific intracellular membrane fractions. Here, we demonstrate that endogenous IP_3_R activity in isolated ER fractions is modulated by the IP_3_R-modulating protein, Bcl-2^48,49^.

The proteins of the Bcl-2 family are known as important players in oncogenic transformation, due to their ability to regulate the balance between cellular death and proliferation^50,51^. Besides their mitochondrial function related to the scaffolding and the inhibition of pro-apoptotic Bcl-2-family members^52^, anti-apoptotic Bcl-2 proteins execute part of their function in the ER through Ca^2+^-signaling modulation by directly targeting and inhibiting IP_3_Rs^53–55^. The molecular determinants underlying IP_3_R/Bcl-2-complex formation have been well characterized^49,56–58^. They are known to involve a Bcl-2-binding site located in the central modulatory domain of the channel^49^ and the BH4 domain of Bcl-2, that is both necessary and sufficient for binding and inhibiting IP_3_R activity^56,57^. A peptide corresponding to the stretch of 20 amino acids of the IP_3_R at this binding site, here referred to as Pep2, is able to antagonize Bcl-2′s inhibitory action on IP_3_Rs^49^.

**Figure 5:**
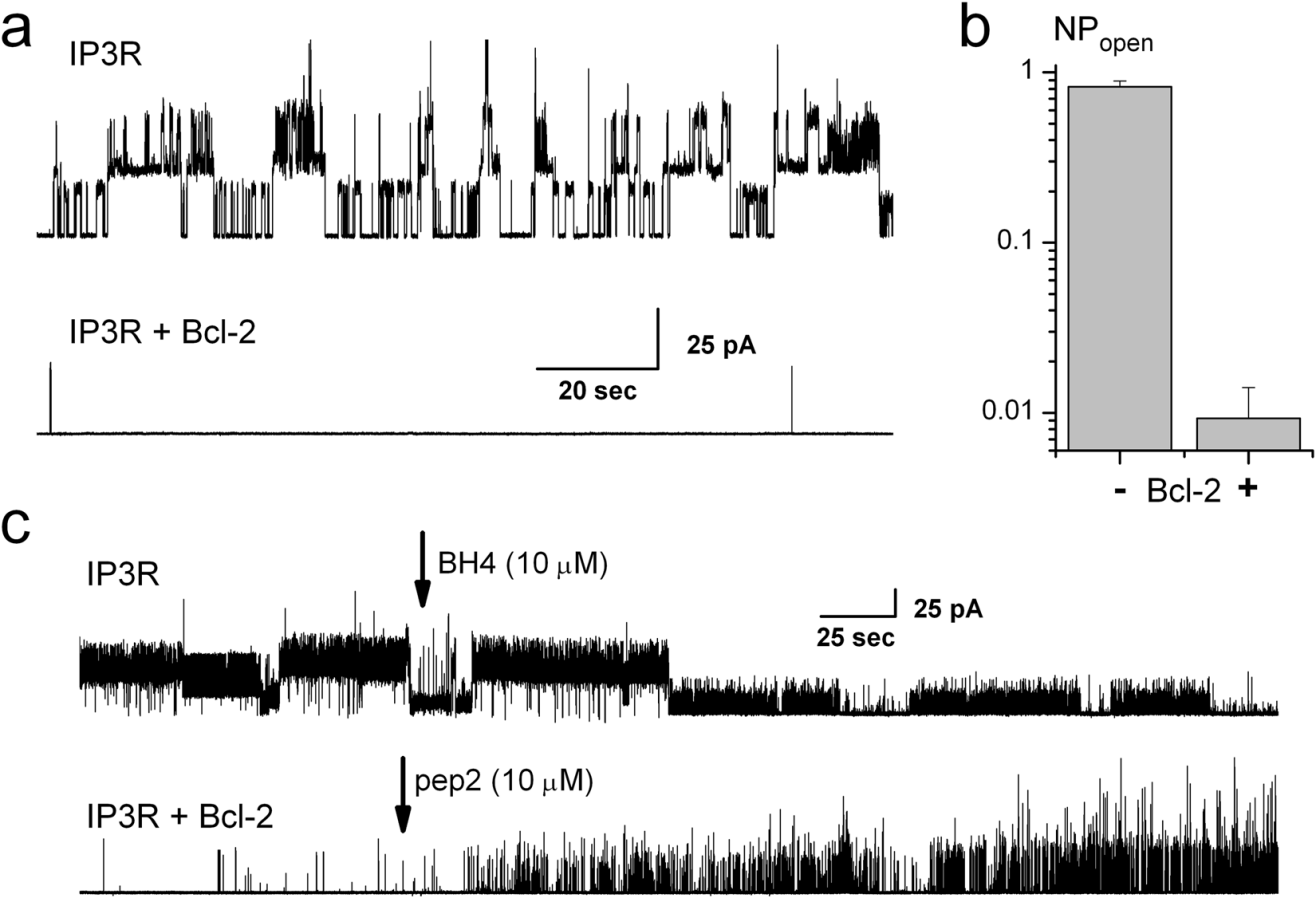
Isolated ER fractions retain interaction of endogenous proteins: activity of IP_3_R compared in control and Bcl-2 expressing WEHI7.2 cells. (a) Recording of IP_3_R gating in control and Bcl-2 expressing cells shows a significant decrease in its activity in the presence of Bcl-2, as summarized in the barplot in (**b**) (mean ± s.e.m.; ***Denotes significant difference with p < 0.001; n = 11 and 12). (**c**) Sample IP_3_R activity in control WEHI7.2 cells can be inhibited by addition of a diffusible fragment of Bcl-2, BH4 (upper panel), while inhibition of IR3P activity in the Bcl-2 expressing cells could be reversed by a diffusible Bcl-2 inhibitor, pep2 (lower panel); n = 7 and 8 correspondingly.

To demonstrate that the presented technique preserves the interaction of intracellular ion channels with their partner proteins, we recorded activity in the GUVs prepared from the ER-containing membrane fractions from control and Bcl-2 ─ overexpressing WEHI7.2 cell lines^57^. The activity was compared between the control (i.e. those expressing only IP_3_R) and Bcl-2 overexpressing variants of these cell lines. As can be seen in Fig. 5a, robust IP_3_R activity could be observed in the GUVs prepared from the ER membrane fractions of the control cells, under the conditions stimulating IP3R (symmetric 1 μM Ca^2+^ and 2 μM IP3). However this activity was significantly less in the GUVs made from the WEHI7.2 cells ectopically expressing Bcl-2, thereby directly confirming the inhibitory effect of the endogenous Bcl-2 on the IP_3_R activity^49^. The average NP_open_ representing levels of activity in the control and Bcl-2 expressing cells are presented in the barplot in Fig. 5b.

To further demonstrate that the interaction between endogenous IP_3_R and Bcl-2 is preserved by our method, we studied the effects of diffusible proteins affecting IP_3_R and Bcl-2 function. As evidenced in the upper panel of Fig. 5c, the IP_3_R activity in the GUVs made from control cell ER fractions could be acutely suppressed by the application of 10 μM BH4-Bcl-2 - a diffusible synthetic peptide representative of the IP_3_R-binding domain of Bcl-2^59^. Finally, and most importantly, the inhibition of IP_3_R activity in the GUVs created from the ER fractions of the Bcl-2 expressing WEHI7.2 cells could be reversed online by the application of 10 μM Pep2 peptide, that targets Bcl-2’s BH4 domain, thereby disrupting the interaction between Bcl-2 and IP_3_R^56,57^.

## Discussion

Understanding the function of ion channels and the roles they play in cellular physiology has long been a subject of numerous studies. The first attempts to register their activity go as far back as 1939, when action potential was for the first time measured in the giant squid axon^60,61^. By the 1970s, even before the advent of the patch-clamp technique, the notion of ion channels as specialized proteins possessing a conductive pore and having the specific properties of ionic permeation, selectivity and gating was already well-established. The same period saw the first observations of the activity of individual ion channels as discrete current levels measured in lipid bilayers upon the addition of “excitability-inducing material” from cellular lipid extracts (e.g., Ehrenstein *et al*.^49^). However, these initial studies were not very specific in targeting particular classes of ion channels and could study only the rudimentary characteristics of these proteins. The development of a patch-clamp technique soon thereafter^62^ presented a robust and easily reproducible way to investigate the properties of ion channels in much greater details, opening a slew of new possibilities to study extensively their function and physiological roles. It is, therefore, quite natural that the majority of ion channel studies quickly shifted towards using this technique, which then became a golden standard of ion channel investigation.

One drawback of the classic patch-clamp, though, is that it is primarily applicable to the ion channels localized in the plasma membrane. Nevertheless, considering the immense array of PM-bound ion channels that had yet to be characterized at the time, this issue was not perceived as a limitation for quite a while. In fact, even the idea that the intracellular ion channels could play important physiological role was not seriously considered up until the identification of IP3R and RyR channels. These channels are now known as “the classical intracellular Ca^2+^-release channels”, serving as major Ca^2+^-release pathways from the ER and playing important roles in regulating apoptosis, autophagy^63–65^, excitation-contraction coupling^2,3^, etc.

Attempts to use biophysical preparations that would allow a study of intracellular ion channels were made as early as 1970s or 80 s, even though at the time they were not specifically targeted at these channels. Commonly, such preparations would involve the fusion of the membrane material with small uni-lamellar vesicles of sub-μm diameter and their further processing via freeze-thaw or dehydration-rehydration cycles, sometimes including the induction of blister formation in order to produce substrates sufficiently large for patching^1^. However, these publications, including authors’ own prior work (e.g. Clayton *et al*. or Shapovalov *et al*.^18,19,66^), typically touched upon procedures to study a particular ion channel under specific conditions, without proposing an approach that would be applicable routinely and universally. With the development of commercial electrophysiological instrumentation and patch-clamp becoming a de-facto standard technique, publications utilizing such strongly biophysical manipulations have been gradually displaced by approaches using either patch-clamp for the PM-bound channels or by studies of the purified intracellular channel proteins (primarily IP3R and RyR) incorporated into planar lipid bilayer. The latter, while allowing a study of intracellular ion channels in a universal way, usually relies on the prior ion channels purification, which translates into a need for large amounts of channel protein. This often requires the use of bacterial preparations and brings with it the associated risk of improper modifications occurring during protein production, especially in the case of eukaryotic channels. Even more importantly, the inherent loss of proteins naturally expressed in the membranes containing the investigated ion channels, makes it impossible to study their regulation by endogenous partner proteins and, thus, limits the applicability of this technique. Some attempts to alleviate this have been made over recent years, e.g. using isolated microsomes to introduce ion channels into the bilayer^19^. While this allows avoiding the protein purification and requires less material in general, this approach, relying on a specific isolation procedure, did not appear to be universaly applicable.

Another way to tackle this problem, which was developed in parallel and presented in scientific literature over time, is patching isolated intact organelles. Indeed, the ability to study ion channel activity under quasi-physiological conditions would yield the most reliable information. One arguably important but not critical limitation of this approach is inability to modify the composition of the studied membranes. A much more important issue, however, is that even now, after many years of intense research of intracellular ion channels, only the nuclear envelope, mitochondria, some plant vacouolar organelles and processed lysosomes have been successfully isolated and patched, largely due to the non-localized nature and fragility of the majority of the important intracellular organelles^67–69^. Thus, neither the regions of ER, apart from that immediately adjacent to the nuclear envelope, nor mitochondria-associated membranes, Golgi, autophagosomes and many other intracellular membranes have been previously patched.

This situation has prompted us to develop a more universal and readily reproducible approach for electrical recording of the activity of intracellular ion channels at a single channel level. This approach builds on a prior work of the first author using an earlier set of biophysical manipulations discussed above^21,23–27,29^ and represents our effort over the years to critically analyze, synthesize, systematize and unify these manipulations. This has resulted in a universal framework of manipulations that a) are applicable universally to a variety of cellular membranes, b) preserve protein contents of investigated membrane(s), thus allowing an investigation of the roles of protein-protein interraction in various signaling cascades recruiting ion channels, c) are versatile, by allowing, if necessary, the modification of protein and/or lipid composition of investigated membrane(s) and d) are simple to implement. This approach has already been successfully used to study the activity of the intracellular isoform of the TRPM8 channel (see Bidaux *et al*.^18,19^ for the characterization of the channel gating, but not the detailed description of the method).

To highlight the universality of our approach, we acquired the activity of IP_3_R and RyR channels from the ER membrane^6^ fractions of the HEK, LNCaP and WEHI7.2 cells, and patched lysosomal fraction of HEK293 cells, showing a characteristic activity of the TPC2 channels, known to be endogenously present in HEK293 cells and to be localized in lysosomal membranes^1^. Finally, we have demonstrated that our approach preserves not only the presence of the endogenous regulatory protein Bcl-2^45,47^, but also its interaction with IP_3_R. Indeed, IP_3_R exhibited a significantly smaller open probability in the cells ectopically expressing Bcl-2 than in the cells void of Bcl-2. Moreover this low P_open_ of IP3R in the presence of Bcl-2 could be restored to control levels by acute application of the pep2 (renamed as IDP in more recent publications) – the peptide inhibiting interaction of Bcl-2 with IP_3_R^56,57^.

It is interesting to compare these results with previous publications reporting the direct effect of Bcl-2 on the IP3R activity. Unlike the Bcl-Xl protein that has been shown to sensitize IP3R to Ca^2+^ at low IP3 concentrations^49^, Bcl-2 has been shown previously to suppress IP3R activity^70^. These publications, however, did not report upon the regulation of IP3R by endogenous Bcl-2, demonstrating instead an effect of either a short soluble fragment of Bcl-2^49,71^ on the activity of IP3R in a nuclear envelope, or the effect of purified full-length Bcl-2 on IP3R reconstituted in planar lipid bilayer^71^. The former publication did not use a full-length Bcl-2 protein, while the latter exhibited a discrepancy commonly observed in studies of the activity of the purified IP_3_R isoforms in bilayer experiments, i.e. clearly different kinetic properties (namely, significantly smaller basal P_open_ in the same set of conditions^49^) compared to the recordings of the IP_3_R activity in natural environment in nuclear envelope. In contrast, our approach allowed successful recording and characterization of the activity of IP_3_R in natural lipid/protein environment, as can be judged from high-P_open_ basal activity of IP_3_R. Furthermore, it revealed for the first time a significantly more complete and robust inhibition of this activity by an endogenous Bcl-2.

Another example of the preservation of protein-protein interactions in our experimental approach, albeit not as direct, is our recording of RyR activity and its sensitivity to dantrolene. In the literature, the effect of dantrolene is controversial. Some publications, primarily using Ca^2+^ imaging and physiological assays, report upon RyR being sensitive to dantrolene^10,49^, while others, in majority utilizing lipid bilayer recordings, report RyR currents to be insensitive to dantrolene^72,73^. Recent publications shed light on this controversy by demonstrating that RyR sensitivity to dantrolene depends on the presence of calmoduline, which is lost in lipid bilayer preparations^68,74,75^. In close correspondence to these data, our approach preserves endogenous protein bundles, thus retaining original protein-protein interaction and thus the effect of dantrolene on RyR activity.

The described procedure not only provides universal means for studying intracellular ion channels while preserving their regulation by partner proteins, but also exhibits a large degree of versatility. The investigator can employ cells overexpressing the ion channel of interest, maximizing the amount of the studied protein, or cell lines naturally expressing investigated proteins, thus enabling studies in cells belonging to physiologically relevant tissue types. Furthermore, one of the principal advantages of this technique - the ability to study the interaction of intracellular ion channels with natural protein partners, can be further expanded by allowing an addition of the membrane-bound factors at the stage of vesicle preparation, or diffusible factors during the data acquisition. It should be noted, that neither retaining protein-protein interaction nor such versatility come at the cost of more tedious manipulations. To the contrary, only the pellet containing the lipid/protein mix corresponding to target organelle(s), and not the isolation of intact organelles, is needed. This allows the use of simpler extraction protocols, while significantly expanding the range of applicable extraction procedures. Any established protocol of (sub)cellular membrane isolation can be used as a part of this approach, allowing one to study a very large selection of organelles. On the other hand, such nature of the employed isolation methods does not provide an easy means to control the orientation of the ion channels in the prepared GUVs. While this may seem to be an essential limitation, in practice, the in-patch orientation of the channel proteins can easily be established in cases when it plays a significant role via the nature of its asymmetry e.g in the case of voltage or ligand-sensitive ion channels, by simply recording channel activity at series of different potentials, or by applying specific agonists to either side of the membrane.

Additionally, compared to the bilayer technique, the ability to use the regular patch-clamp systems allows the acquisition of higher-resolution and lower-noise data, which is essential in building the detailed kinetic and molecular models of the regulation of intracellular ion channels. Finally, this procedure naturally combines with the use of the automated patch-clamp systems. Thus, a significant portion (approximately 40%) of the measurements reported in this publication were performed using the Port-a-Patch automated patch system by Nanion.

In conclusion, intracellular ion channels form a major class of ion channel proteins, likely even more numerous than the “classic” PM-localized channels, and regulating important processes such as the balance between cellular proliferation and death or cellular motility and cancerogenesis. However, in-detail functional studies of these ion channels, especially at single-channel level, are still not very common due to the disparity of the currently available experimental techniques. Especially lacking is the ability to study ion channels localized in arbitrary intracellular organelles while maintaining their interaction with endogenous partner proteins. Here, we present a streamlined universal approach, named organelle membrane derived (OMD) patch clamp that addresses these limitations, exposing the intracellular ion channels to routine functional studies and allowing extensive investigation of their regulation and role in cellular signaling.

## Materials and Methods

### Membrane proteins extraction

Cells were harvested with a rubber scraper in a phosphate buffer saline solution (PBS), pelleted in 50 ml tubes and homogenized in ice-cold buffer B (250 mM sucrose, 1 mM EDTA, 20 mM Hepes-NaOH pH 7.4 and a mixture of protease inhibitors (P8340 by Sigma-Aldritch)) using a glass potter homogenizer. Non-disrupted cells and the post-nuclear fraction were pelleted twice consecutively at 250 ***g*** for 15 min at 4 °C, prior to being re-suspended in solution B and spun at 1,000 ***g*** for 15 min at 4 °C in order to separate whole cells in the bottom pellet and the post-nuclear fraction in the top pellet. Crude mitochondria extract, the supernatant, was centrifuged at 17,000 ***g*** for 30 min at 4 °C in order to pellet mitochondria and associated membranes. The supernatant was completed with 1 volume of Hepes buffer (20 mM Hepes-NaOH pH 7.4, 1 mM EDTA and a mixture of protease inhibitors) and proteins were pelleted at 100,000 ***g*** for 1 h at 4 °C on a 50Ti rotor before re-suspension in 1 mL ice -cold solution B. The remaining supernatant, after microsome pelleting, was precipitated in 5 volumes of ethanol overnight at −20 °C. The crude mitochondria pellet was processed on an OptiPrep™ Density Gradient Medium (DGM) ranging from 10 to 30% of iodixanol prepared as explained on the datasheet. After centrifugation at 100,000 ***g*** for 2 h at 4 °C on a SW40 rotor, 4 fractions were isolated and further subjected to a 1 h centrifugation at 100,000 ***g*** at 4 °C on a 50Ti rotor.

Each final pellet was either re-suspended in a RIPA buffer (10 mM PO4Na2/K buffer, pH 7.2, 150 mM NaCl, 1 g/100 ml sodium deoxycholate, 1% Triton X-100, 1% NP40, a mixture of protease inhibitors (Sigma-Aldrich), and a phosphatase inhibitor (sodium orthovanadate; Sigma-Aldrich)), or briefly vacuum-dried to remove the aqueous component, followed by re-suspension in 5:1 CHCl_3_/MeOH (chloroform/methanol) solution at saturating concentration (typically ~1 ml of CHCl_3_/MeOH per protein yield from 1000,000 cells). Protein titration of RIPA-suspended extracts was performed with BCA protein assay (Pierce).

### Preparation of patchable substrates (GUVs)

The isolated membrane fractions were combined with an appropriate collection of lipids dissolved in chloroform or 5:1 CHCl_3_/MeOH solution. Typically, a good yield of patchable vesicles could be produced when utilizing the combination of diphytanoylphosphatidylcholine (DPhPC), 1-palmitoyl-2-oleoyl-sn-glycero-3-phosphocholine (POPC) and cholesterol with the ratios of 9:0:1 or 7:2:1. Lipids and fractions were diluted as necessary and mixed, with a straight 1:1 combination of 5 mM lipids and saturating suspension of subcellular membrane fraction commonly leading to the observation of multiple IP3 channels per patch upon stimulation with IP3. In order to achieve a density of only a single channel per patch, a further dilution of the isolated fractions can be performed by adding more lipids. Typically, the mixtures with 1:2 – 1:5 ratios were used (e.g. for material extracted from HEK293 and WEHI7.2 cells correspondingly), yielding activity of IP3R channels in approximately half of the patches.

The addition of essential lipid factors (e.g. PIP_2_, which is known to regulate many ion channels) is possible at this stage if the studied proteins require it. Proper uniform mixing of the proteins and lipids was ensured by rigorous vortexing (30 sec at maximum speed) and sonication of the glass vial with the mixture in a bath sonicator (600 W) for 15 min. In the case of membrane fractions solubilized in a detergent-containing buffer, the decided-upon amount of lipids, conforming to the selected ratio, as discussed above (e.g. 10 μl of 5 mM lipids for each 10 μl of solubilized membrane fraction, corresponding to a 1:1 ratio) was deposited in an eppendorf tube and air-dried. The matching amount of membrane fraction material still in detergent buffer was deposited in the tube and the contents vortexted and sonicated (e.g., 600 W sonicator for 15 min). The detergent was then removed by adding 50% by volume of SM-2 resin Biobeads (Bio-Rad, Hercules, CA, USA) overnight on a rotary stage. This resulted in the formation of SUVs visible as a light nebulous substance formed upon detergent removal. The volume contained in this material was then collected for further processing, in order to form GUVs.

To prepare giant uni-lamellar vesicles (GUVs), 10 μl of the final mixture were dried on a clean ITO glass slide. In the case of the SUV deposit (when using the membrane fraction material solubilized in detergent-containing buffer), drying of the mixture was performing under house vacuum, followed by an application of 10 μl of chloroform in order to uniformly redistribute the protein-containing lipids on the ITO glass slides and another drying. This was followed by rehydration of the formed film with 250 μl of 300 mM sucrose or sorbitol solution in the space enclosed by the rubber o-ring and covered by another ITO glass slide. The completed chamber was positioned in a Vesicle Prep Pro apparatus (Nanion Technologies, Germany) and GUVs were produced utilizing the basal electroformation protocol^32^, that applied 3 V with the frequency of 5 Hz for 110 min with 5 min periods before and after the main run, when the voltage was gradually increased and decreased from/to 0 V. Produced GUVs were collected immediately after protocol completion (GUVs are unstable in contact with glass) and transferred into an eppendorf tube. Formed GUVs could be utilized for recordings immediately or stored for up to 1 week at 4 °C.

### Western-blot

25 µg of gradient-purified fractions were loaded onto a Bolt^TM^ 4–12% Bis-Tris Plus gels using a Mes buffer (Invitrogen), according to the manufacturer’s instructions. After wet electrophoretic transfer onto a Polyvinylidene Difluoride (PVDF) membrane, the membrane was blocked in a PBS containing 0,1% Tween 20 (v/v) and 5% (w/v) non-fat dried milk for 1 hour at room temperature, then soaked in a primary antibody diluted in PBS + 0,1% Tween 20 + 1% milk for 1 hour at room temperature. After three washes in PBS + 0,1% Tween 20, membranes were soaked in a secondary antibody diluted in PBS + 0,1% Tween 20 + 1% milk for 1 hour at room temperature. Membranes were processed for chemiluminescence detection using SuperSignal^TM^ West Dura Extended Duration Substrate (Thermo Scientific), according to manufacturer’s instructions. The immuno-reactive bands were visualized using the Amersham Imager 600 (GE Healthcare Life Sciences).

The primary antibodies were: mouse VDAC1, 1:200 (Santa Cruz Biotechnologies, Inc., SC-58649), mouse anti-Golgin-97, 1:1000 (Molecular Probes®, A21270) and mouse anti-Calnexin, 1:3000 (Millipore, MAB 3126) and 1:1000 polyclonal rabbit anti-IP_3_R Ab Rbt475 (recognizing all three IP_3_R isoforms; a kind gift of Dr J.B. Parys^30^).

### Patch clamping

Patch-clamp experiments were performed using an Axopatch 200B amplifier and pClamp 10.0 software (Molecular Devices, Union City, CA) for data acquisition and analysis. Patch pipettes were fabricated from borosilicate glass capillaries (World Precision Instr., Inc., Sarasota, FL) on a horizontal puller (Sutter Instruments Co., Novato, CA) and had a resistance in the range of 7–10 MΩ.

Recordings were carried out in patches made of prepared GUVs. The eppendorf tube, where vesicles were kept, was slightly shaken to uniformly distribute GUVs and then vesicles were transferred to the bath containing, typically, KCl-based solution (in mM: 150 KCl, 5 glucose, 10 MOPS, pH 6.5, adjusted with KOH). Patch pipettes were, commonly, filled with an equivalent solution. This base solution could easily be changed to one based on a different cation or even a solution with a higher salt content, as long as the corresponding change to sucrose/sorbitol osmolarity is carried out during the GUV preparation. For the purpose of the described experiments, the changes in the base solution involved only control of Ca^2+^ and Mg^2+^ concentrations and the addition of 1 mM of K_2_ATP to the pipette. Specifically, to emphasize the activity of IP_3_R channels^39^, free Ca^2+^ was clamped at ~0.5 μM by supplementing the base solution with 50 μM CaCl_2_ and 1 mM EGTA. Further, 2 mM MgCl_2_ were added to suppress RyR activity. IP_3_R activity was stimulated by the addition of 10 μM IP3. During the recordings of the RyR activity, base solution was only supplemented by the CaCl_2_/EGTA (50/40 μM) combination to clamp free Ca^2+^ at ~30 μM, omitting MgCl_2_ addition. Spontaneous RyR activity, observed under such conditions, was further stimulated by the addition of 3 mM caffeine and blocked by 1 mM dantrolene, while 1 nM dantrolene increased the Popen of the channel^10^.

Typically, approaching the GUV with a clean pipette and without applied pressure or suction led, upon touching the vesicle, to immediate gigaseal formation and bursting of the GUV. Following this, the recording could be commenced at any point as in regular electrophysiological experiments in excised patch configuration. Recorded activity was quantified by performing a single-channel search analysis using the Clampfit-10 (pClamp software suit, Molecular Devices, Sunnyvale, CA, USA) and QuB 2.0 programs. Statistical analyses, t-tests and Gaussian fits to amplitude distributions were carried out using Microcal Origin software. Values are expressed as mean ± s.e.m., unless indicated otherwise.

## Electronic supplementary material

Supplementary Figures
