# Supplementary material for: Organelle membrane derived patches: reshaping classical methods for new targets
Source: Sci Rep. 2017 Oct 26;7:14082. doi: 10.1038/s41598-017-13968-y (PMC5658434; doi:10.1038/s41598-017-13968-y)
Supplement: Supplementary file 1 — Supplementary Figures [file 41598_2017_13968_MOESM1_ESM.pdf]

Supplementary Information

Organelle membrane derived patches: reshaping classical methods for new targets

George Shapovalov[12], Abigaël Ritaine[12], Gabriel Bidaux[123], Christian Slomianny[12], Anne-Sophie Borowiec[123], Dmitri Gordienko[12], Geert Bultynck[4], Roman Skryma[125], Natalia Prevarskaya[125*]

[1] Inserm U1003, Equipe Labellisée par la Ligue Nationale Contre le Cancer, Université de Sciences et Technologies de Lille (USTL), F-59655 Villeneuve d'Ascq, France

[2] Laboratory of Excellence, Ion Channels Science and Therapeutics; Université Lille I Sciences et Technologies, Villeneuve d'Ascq, France

[3] Present adress : Laboratoire INSERM U1060, CarMeN Laboratory, Claude Bernard Lyon 1 University, 8, avenue Rockfeller, F-69373 Lyon, France

[4] KU Leuven, Laboratory of Molecular and Cellular Signaling, Department of Cellular and Molecular Medicine, Herestraat 49, BE-3000, Leuven, Belgium

[5] Shared senior authorship

George Shapovalov and Abigaël Ritaine contributed equally to this work.

* Correspondence and requests for materials should be addressed to: natacha.prevarskaya@univ-lille1.fr

Supplementary Figure S1. Original (uncut) gels used for membrane fraction attribution. Related to Fig. 2, which shows the same data rearranged for clearer presentation.

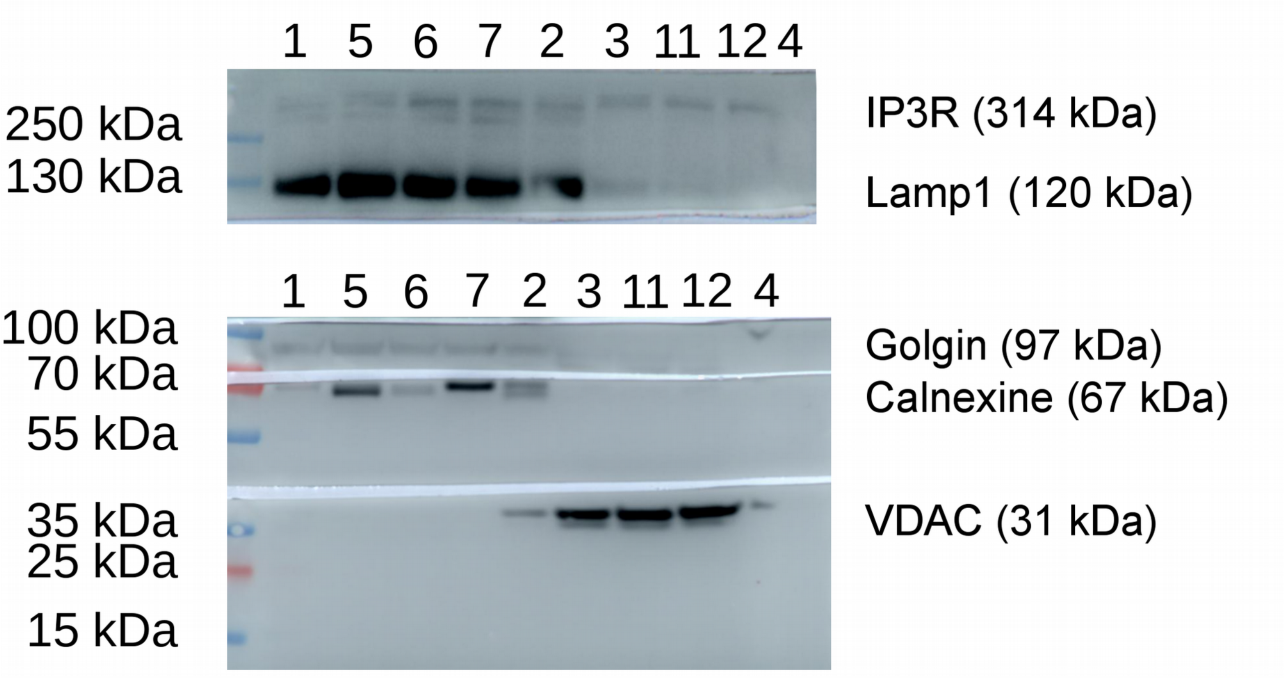

Original, uncut gels representing Western blot characterization of as IP3R presence (top) as well as organelle-specific markers (bottom) in the isolated fractions following single- (b) and double- (c) gradient isolation of subcellular membrane fractions, as indicated on diagram in Fig 2a. ER membranes are reported by the detection of calnexin (upper panels), while Golgi apparatus and mitochondria are characterized by the detection of golgin-97 and voltage-dependent anion channel 1 (VDAC1) (middle and lower panels), respectively. Number of the corresponding isolated membrane fraction is shown at the top of each gel. Note that, following the double-gradient isolation, Fractions 8, 9 and 10 did not contain sufficient amounts of protein and were thus not characterized. Experiments were reproduced 4 times independently.

Supplementary Figure S2. RyR activity in whole-membrane extracts from HEK293 and in ER fractions from LNCaP cells. Related to Fig. 3.

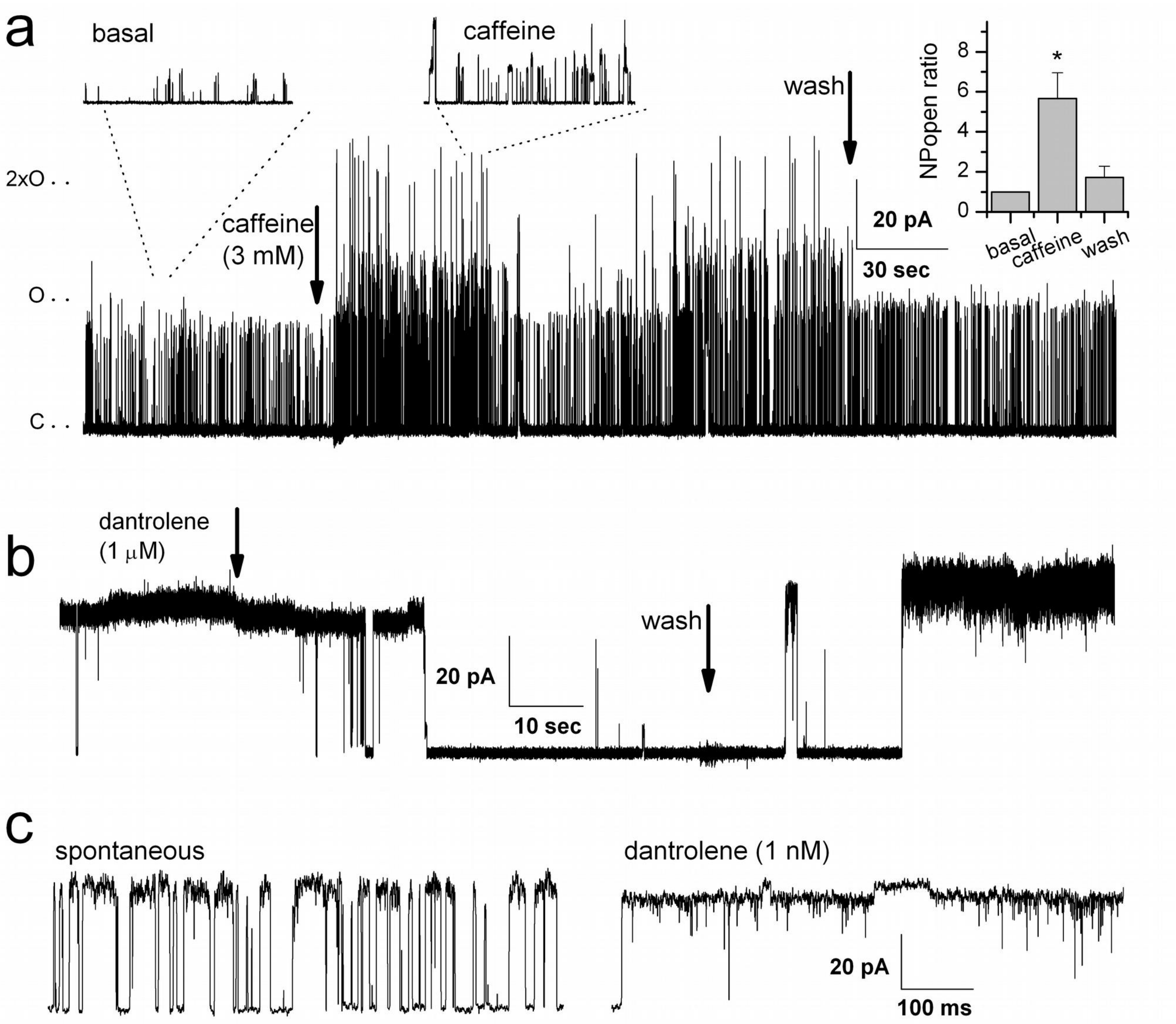

Membrane fraction extracts, that were prepared as described, were mixed with DPhPC/10% cholesterol lipid mixture and used for GUV preparation. Activity was recorded as described elsewhere in the article with base bath/pipette solutions containing 150 mM KCl, 10 mM MOPS, pH 6.4. To enhance RyR activity, pipette and bath solutions contained no additional Mg^{2+} and had Ca^{2+} stabilized at 30 μM by addition of an appropriate Ca^{2+}/EGTA combination (0.5 and 0.4 mM correspondingly at T=22ºC and pH=6.4). Under such conditions, spontaneous RyR activity could be observed in, approximately, 1 out of 3 patches.

(**a**) Sample RyR activity at Vm=80 mV, showing basal region followed by stimulation with 3 mM caffeine and, finally, wash-out. Insets on the left show higher resolution fragments of traces in basal and caffeine-stimulated regions. Note the characteristic presence of multiple conductive substates as well as apparent increase of open dwell times following the stimulation by caffeine. Barplot inset on the right summarizes stimulation of spontaneous activity by caffeine (n=4, mean ± s.e.m.; * denotes significant difference with $p<0.05$). (**b**) Inhibition of RyR activity by dantrolene. In all traces application of high concentrations (1 μM) of dantrolene led to complete suppression of activity in a reversible manner. (**c**) Application of low concentration (1 nM) of dantrolene increases Popen during single-channel gating of RyR.
